# Is Retinol Binding Protein 4 a Good Biomarker of Renal Function in Children with Neurogenic Bladder After Myelomeningocele?

**DOI:** 10.3390/jcm14072520

**Published:** 2025-04-07

**Authors:** Alicja Szymańska, Joanna Bagińska-Chyży, Agata Korzeniecka-Kozerska

**Affiliations:** Department of Pediatrics and Nephrology, Medical University of Białystok, 17 Waszyngton Str., 15-274 Białystok, Poland; iklinped@umb.edu.pl (A.S.); joasiabaginska14@wp.pl (J.B.-C.)

**Keywords:** neurogenic bladder, renal function, biomarker

## Abstract

**Background:** This prospective study aimed to evaluate renal function using retinol binding protein 4 (RBP4), cystatin C, and glomerular filtration rate (GFR) in relation to physical activity and lesion level in children with neurogenic bladder (NB) post-myelomeningocele. **Methods**: Two groups were studied: 33 children with NB and 20 healthy controls. Data collected included demographic details, physical activity levels, uroflowmetry, urodynamic diagnosis, and renal function parameters. Urinary RBP4 and serum cystatin C were measured using ELISA, and GFR was calculated using the Schwartz formula. **Results**: The NB group had higher median serum cystatin C and urinary RBP4/creatinine ratios compared to the control group (0.28 vs. 0.22; 18.6 vs. 3.2, respectively). The participants were categorized based on activity levels, lesion levels, catheterization status, and urodynamic diagnosis. No differences in RBP4, cystatin C, or urodynamic diagnosis were observed according to activity and lesion levels. Significant differences in GFR were found based on activity and lesion levels, with higher median GFR in NB children (182.7 vs. 147.3). No differences were found between catheterized and non-catheterized children in the studied parameters. **Conclusions**: Elevated urinary RBP4 in NB patients suggests possible proximal renal tubule dysfunction. Higher serum cystatin C despite lower creatinine levels indicates altered renal function in NB children. Urinary RBP4 correlates positively with bladder pressure at maximum cystometric capacity, suggesting potential utility in therapy monitoring and modification.

## 1. Introduction

Myelomeningocele (MMC) is a severe congenital malformation occurring in approximately 1 per 2000 live births [1]. Clinical examination of these patients often reveals paralysis, hydrocephalus, and various malformations of the nervous system. Additionally, they frequently suffer from undetected renal function impairment due to neurogenic bladder, which can lead to chronic kidney disease and progress to end-stage renal disease, necessitating renal replacement therapy. Effective management of children with MMC requires a multidisciplinary approach involving a neurosurgeon in the early days of life, followed by ongoing care from a neurologist, a rehabilitation team, and consistent monitoring by an experienced nephrologist. Renal damage in MMC patients is caused by mechanisms similar to those in obstructive uropathies. Notably, children with MMC constitute a substantial proportion of pediatric dialysis patients, with research showing that 15% of those on dialysis have neurogenic bladder [2,3,4].

Urodynamic investigations in children with neurogenic bladder post-MMC reveal various dysfunctions, including overactive bladder and/or underactive detrusor and sphincter abnormalities. A lot of these patients require pharmacological correction of the detrusor function and/or sphincter, and most of them need clean intermittent catheterization (CIC) and very frequent estimation of kidney function. Pharmacological treatment is guided by urodynamic data and typically includes the standard approach of CIC. The chosen treatment aims to enhance urinary bladder function and achieve social urinary continence, which are critical for the well-being of patients. According to the principle that prevention is better than treatment, it is crucial to identify early markers of urinary bladder and kidney damage in children with MMC to reduce the incidence of chronic renal failure. [5]. At birth, the majority of patients have normal upper tracts, but nearly 60% of them will develop upper tract deterioration due to increased detrusor filling pressures and infections, with or without refluxes [6].

Initially, the primary objective of therapy was the long-term preservation of renal function, with early detection yielding the most favorable outcomes for maintaining kidney health [7]. Creatinine is the most commonly utilized biomarker for assessing kidney function. However, it is not reliable for detecting mild renal impairment, as its levels can vary with muscle mass. Serum cystatin C levels provide a more accurate measure of kidney function compared to serum creatinine concentrations [8]. Urinary excretion of retinol binding protein 4 (RBP4) is emerging as a sensitive indicator of renal tubular damage. Elevated levels of urinary RBP4 can reflect dysfunction in the proximal renal tubules, even before significant changes in GFR or serum creatinine are detectable [9].

To the best of our knowledge, our study represents the first investigation of the relationship between RBP4 and the renal function of patients with MMC. The detailed research goals include the following:

1. To compare urinary RBP4 concentration between children with MMC and healthy participants.
2. To establish the relationship between urinary RBP4 concentration in MMC and compare it with markers of renal function, as well as urodynamic and biochemical parameters.

## 2. Materials and Methods

The prospective investigation was conducted on 53 children, divided into two groups. The first group constituted 33 children (group 1) with a median age of 8.8 years (1.83–18) who were operated on for MMC in the neonatal period and diagnosed with neurogenic bladder based on urodynamic investigations. Urinary tract infections were excluded on the basis of urinary testing. The negative C-reactive protein result excluded the current inflammation. We divided the MMC patients according to motor activity into 4 groups (based on Hoffer’s scale: group 1—non-ambulator, group 2—nonfunctional ambulator, group 3—household walkers, group 4—community walkers) and lesion level into 3 groups (group 1—Th-L—thoraco-lumbar level, group 2—lumbar level, group 3—sacral level).

The second group comprised 20 healthy children (group 2, median age: 10.6 years, range: 1–17) with no urinary or neurological abnormalities, confirmed by normal urine tests and CRP levels).

A first-morning urine sample and a 24-h collection were obtained from each patient to measure urinary RBP4 concentrations. The 24-h urine samples were kept at +4 °C during collection and were frozen immediately after completion. The samples were stored at −80 °C to prevent degradation before analysis. The period of urine sample collection was 6 months, which was the duration of the project. After collecting all samples, the RBP4 was estimated according to the manual instructions. The estimation of RBP4 using an ELISA set typically follows these steps: the ELISA plate is pre-coated with an antibody specific to RBP4. Standards, controls, and urine samples are prepared and diluted as needed. Standards and test samples are added to the wells of the ELISA plate. The RBP4 in the samples binds to the immobilized antibody. A biotinylated detection antibody specific to RBP4 is added to the wells. This antibody binds to the captured RBP4, forming a “sandwich”. Streptavidin conjugated to an enzyme is added. It binds to the biotinylated detection antibody. A substrate solution is added, which reacts with the enzyme to produce a color change. The intensity of the color is proportional to the concentration of RBP4 in the sample. The reaction is stopped using an acidic solution, and the color intensity is measured using a microplate reader at a specific wavelength. A standard curve is generated from the known concentrations of RBP4 standards, and the sample concentrations are calculated based on this curve.

Renal function was estimated using the Schwartz formula based on serum creatinine levels and cystatin C. Additionally, the osmolality of urine was estimated in all patients from the first portion of urine (after the night) following a previous twelve-hour liquid limitation. We obtained consent from the patients and their parents for all procedures. The urodynamic investigations were performed on patients with MMC after typical preparation. The following parameters were analyzed: the detrusor pressure at urgency (P det urg), the detrusor pressure at maximum cystometric capacity (P det CC), the compliance of the bladder wall (Comp), and electromyography (EMG) of the sphincter at the beginning (EMG 1) and at the end (EMG2) of filling phase. The infusion volume was calculated as the average volume of urine obtained from CIC to estimate the bladder function in the most approximate way for everyday filling and emptying of the bladder in natural conditions. Children with MMC who were not catheterized performed uroflowmetry 3 times, and the average values were analyzed. Uroflowmetry was also performed on the healthy children group following the same procedure.

The RBP4 urinary concentration was estimated using the ELISA set. The investigation was executed according to the manual instructions. The total RBP4 urinary level was standardized to the creatinine (assessed in the same samples of urine), and the result was expressed as RBP4/creatinine ratio (ng/mg creatinine).

The results were statistically analyzed using the computer program Statistica 12.0. Since the Shapiro–Wilk test indicated no normal distribution, statistical analysis was conducted using the nonparametric Mann–Whitney test. The Spearman test was used to assess the correlations among the studied parameters. A *p*-value less than 0.05 was considered statistically significant.

## 3. Results

The clinical characteristics of the two studied groups are shown in Table 1.

There were no differences in age (*p* = 0.11), but the differences in the physical development parameters (the body weight *p* = 0.017 and height *p* = 0.001), which were observed, resulted from the principal disease. MMC patients exhibit lower muscle mass due to limb paralysis or increased body weight resulting from reduced physical activity. Moreover, the differences in height are often caused by distortion and malformations of the bone structure.

MMC patients were assessed according to motor activity. Fourteen out of thirty-three (42%) were wheelchair dependent, 5/33 (15%) were nonfunctional ambulators, 4/33 (12%) were household walkers, and 10/33 (30%) were community walkers. Five out of thirty-three (15%) MMC patients present thoraco-lumbar lesion level (Th-L), 21/33 (64%) lumbo-sacral level (L-S), and 7/33 (21%) sacral level.

More than 25/33 (75%) MMC patients needed CIC, and only 8/33 (25%) did not need CIC. No statistical differences were observed in GFR, RBP4, and cystatin C among catheterized and non-catheterized patients (*p* = 0.75, *p* = 0.21, and *p* = 0.26, respectively). We found statistically significant differences in GFR according to Hoffer’s scale in group 1 (193.5 vs. 172 vs. 191.1 vs. 125.2 mL/1.73 m^2^/min; Chi2 = 7.82, *p* = 0.049) and according to the lesion level (197.5 vs. 148.5 vs. 128.7 mL/1.73 m^2^/min; Chi2 = 9.92, *p* = 0.007). There were no differences in RBP4 and cystatin C among patients on different levels of lesion groups (Chi2 = 1.58, *p* = 0.45 and Chi2 = 0.16, *p* = 0.92).

Table 2 includes a comparison of urodynamic parameters between the MMC and reference groups. Statistically significant differences were observed in delay time (T del) and maximum flow rate (Q max) between patients with MMC and the reference group. The voided volume in MMC patients was three times lower than that of the reference group, and post-void residual urine was substantially higher in the MMC cohort. Analysis of urodynamic parameters revealed a positive correlation between RBP4 and Pdet CC (r = 0.35, *p* < 0.05).

We identified a statistically significant correlation between uric acid levels and both cystatin C and creatinine in the MMC group. Table 3 presents the correlations among biochemical parameters, RBP4, and the RBP4/creatinine ratio in MMC patients. Table 4 includes correlations between urodynamic and renal function parameters and the Hoffer scale.

## 4. Discussion

The results of our investigation showed that children with neurogenic bladder after MMC have significantly higher RBP4 urinary levels in comparison to the reference group. Because of essential statistical differences in creatinine concentration in urine and GFR between the two studied groups, we standardized the RBP4 concentration to creatinine. Limited mobility and paralysis in MMC patients can result in low muscle mass, which can subsequently affect creatinine concentrations in both serum and urine. The above raises the question of whether GFR calculated from creatinine level is adequate for MMC children. According to suggestions from many publications, it is crucial to use more detailed and independent markers, such as cystatin C or a more sensitive GFR ratio calculated from the combined creatinine and cystatin C concentration in patients with neurogenic bladder [10,11,12]. However, after almost 70 years of scientific work and more than 70 formulas estimating GFR based on creatinine and cystatin C, there are a lot of doubts regarding the correct estimation of glomerular function [13]. Our study demonstrated that patients with neurogenic bladder exhibit lower serum creatinine concentrations compared to the reference group, alongside higher levels of cystatin C, RBP4, and RBP4 standardized to creatinine excretion in urine. These findings suggest potential kidney dysfunction, likely attributable to proximal renal tubule impairment. MMC patients face an increased risk of progressing to chronic kidney disease, highlighting the critical need for early biomarkers to detect proximal tubule damage and preserve renal function as early as possible. Based on our research, the measurement of urinary excretion of RBP4 in MMC patients appears promising in fulfilling this need.

GFR is regarded as the optimal indicator of renal function in both healthy children and those with kidney disease. The gold standard for measuring GFR involves the urinary or plasma clearance of exogenous filtration markers such as inulin or iohexol. GFR can also be estimated using endogenous filtration markers, specifically serum creatinine or cystatin C. However, as previously mentioned, estimating GFR based on creatinine can be problematic due to factors such as low muscle mass, bone deformities, reduced height, or paralysis [8,14]. It is challenging to determine whether lower creatinine levels and higher GFR values calculated from creatinine indicate hyperfiltration and potential kidney dysfunction or if they are merely the result of methodological bias. When considered alongside elevated urinary RBP4 excretion, it can be inferred that renal function may be compromised in patients with MMC.

In our study, no differences were observed in RBP4 levels and the RBP4/creatinine ratio between catheterized and non-catheterized patients with neurogenic bladder. This finding suggests that despite normal results in non-catheterized patients, kidney function may still be compromised. Conversely, the absence of differences might indicate that CIC was implemented appropriately as bladder function deteriorated. This observation is novel, as there are no similar reports in the existing scientific literature. Our study is pioneering in this area and warrants confirmation in a larger pediatric population.

RBP4 is also recognized as a marker of adequate nutrition, with low serum concentrations indicating malnutrition at various stages [15,16,17,18]. Our study revealed that MMC patients were lighter than their peers in the reference group. It is plausible that increased urinary excretion of RBP4 could contribute to lower serum RBP4 levels. This hypothesis requires validation in a larger population, with additional measurements of serum RBP4 levels.

Urinary RBP4 is an independent predictor of renal outcomes in various diseases. Its unique role is well-documented in diabetic nephropathy, where it serves as an early marker of kidney damage [9,19,20]. Numerous studies have established its significance in acute kidney injury, particularly in assessing the function of the proximal renal tubule. As was proven by Gonzales-Calero L. et al. [21], increased concentrations of RBP4 were detected earlier, before increased levels of creatinine. Based on these reports, it can be asserted that urinary RBP4 levels may serve as an early marker of kidney impairment in patients with neurogenic bladder. This finding potentially provides a partial answer to the question of whether we can predict which patients will develop chronic renal disease.

There are no data in the literature regarding RBP4 urinary excretion in MMC patients and its correlation with urodynamic parameters. In our study, the bladder was filled to a volume equivalent to that used in the patient’s routine catheterization. The positive correlation among RBP4, RBP4/creatinine ratio, and detrusor pressure at maximum cystometric capacity suggests that the frequency of bladder emptying throughout the day may be insufficient despite detrusor pressure at the end of the filling phase remaining within the reference range (not exceeding 20 cm H_2_O). Considerably raised RBP4 values in MMC patients in relation to the reference group may result from neurological damage; however, the estimation of this is very difficult and controversial. On the contrary, in our study, we provide a positive correlation between RBP4 urinary level and detrusor pressure at maximum capacity in the group of studied children. It may be that the values recognized as normal (not exceeding 40 cm H_2_O) should be modified to a much lower number. The urinary RBP4 level may be useful in assessing the storage phase in patients with neurogenic bladder. Elevated intravesical pressure during the filling phase and low bladder wall compliance pose significant risks to the upper urinary tract, potentially leading to obstructive nephropathy, renal failure, and the necessity for renal replacement therapy [22,23].

In our cohort of patients with MMC, no kidney damage was observed. The parameters assessing renal function, including uric acid, creatinine, GFR, cystatin C, and urinary osmolality, were within normal ranges. However, we noted significantly higher levels of cystatin C (*p* < 0.05) in the MMC group, which may be attributed to incomplete or ineffective bladder emptying in these patients. This is supported by the positive correlation between residual urine volume and both RBP4 and the RBP4/creatinine ratio in urine. The limitation of our study is the relatively small sample size, and further larger studies with larger cohorts or longitudinal surveys are required to confirm our results.

Our conclusion is as follows:

1. Urinary RBP4 and RBP4/creatinine ratio in MMC patients are statistically higher compared to the reference group, which can suggest dysfunction in the proximal renal tubule.
2. Serum cystatin C in MMC children is statistically higher compared to the reference group despite the creatinine level being lower.
3. Hyperfiltration in children with neurogenic bladder may be considered a marker of incorrect renal function.
4. The urinary RBP4 level in neurogenic bladder patients showed significant associations with bladder pressure at maximum cystometric capacity, indicating its potential utility in evaluating the storage phase and supporting therapeutic decisions.

## Figures and Tables

**Table 1:** Clinical characteristics and comparison of both studied groups: group 1 (MMC patients) and group 2 (reference group). *p* value * *p* < 0.05; ** *p* < 0.001.

Parameters	Group 1	Group 2	*p*
	Median (Minimum–Maximum)		
Age (years)	8.8 (1.83–18)	10.6 (1–17)	0.11
Height (cm)	120 (81–170)	150 (76–190)	<0.001 **
Body weight (kg)	29 (6.87–92)	36.5 (8.1–71)	0.017
BMI (kg/m^2^)	15.9 (9.08–37.2)	18.1 (12.02–24.5)	0.66
SUA (mg/dL)	3.9 (1.32–7.3)	3.9 (3.35–5.3)	0.74
S creat (mg/dL)	0.3 (0.18–0.8)	0.5 (0.2–0.8)	<0.001 **
U creat (g/24 h)	0.52 (0.16–1.11)	1.3 (0.63–2.44)	<0.001 **
CysC (mg/mL)	0.28 (0.17–0.54)	0.22 (0.07–0.31)	0.039 *
eGFR (mL/min/1.73 m^2^)	182.7(103.9–310)	147.3 (110–197.77)	0.04 *
24-h urine (mL)	660 (100–1500)	925 (400–1500)	0.21
Urine osmolality (mOsm/L)	711.5 (357–1177)	690 (392–1200)	0.74
RBP4 (ng/mL)	0.54 (0.01–2.06)	0.29 (0.06–0.46)	<0.001 **
RBP4/creat (ng/mg)	18.6 (2.49–48.5)	3.2 (1.95–11.5)	<0.001 **

SUA—serum uric acid level; S creat—serum creatinine level; U creat—urinary creatinine level in 24-h collection of urine; CysC—serum Cystatin C level; eGFR—glomerular filtration rate calculated based on Schwartz formula, 24-h urine—24-h collection of urine; RBP4—retinol binding protein 4; RBP4/creat—RBP4/creatinine ratio.

**Table 2:** Comparison of uroflowmetry parameters between MMC patients and reference group and characteristics of cystometric parameters in MMC patients. * *p* < 0.05, ** *p* < 0.001.

Parameters	Group 1	Group 2	*p*
Median (Min–Max)			
Uroflowmetry			
TV (s)	22 (3–95.6)	18 (11–41)	0.98
TQ (s)	16 (2–38.8)	16 (9–38)	0.83
TQ max (s)	9 (1–28)	7 (4–12)	0.33
T del (s)	16 (7–280)	2 (1–13)	<0.001 **
Q max (mL/s)	7 (1.4–30.4)	20.2 (13.3–32)	0.011 *
Q avr (mL/s)	3.1 (0.5–15.7)	-	-
VV (mL)	77.7 (5–133.5)	219 (104–456)	<0.001 **
PVR (mL)	75 (0–100)	0 (0)	<0.001 **
Cystometry			
P det urg (cmH_2_O)	35 (4–100)		
P det CC (cmH_2_O)	13.5 (1–40)		
Max CC (mL)	143 (38–270)		
Comp (mL/cmH_2_O)	11.6 (0.3–50)		
EMG 1 (mV)	2 (0–60)		
EMG 2 (mV)	5 (0–50)		

TV—voiding time; TQ—flow time; TQ max—time to maximum flow rate; T del—delay time; Q max—maximum flow rate; Q avr—average flow rate; VV—voided volume; PVR—post-void residual; P det urg—the detrusor pressure at urgency; P det CC—the detrusor pressure at maximum cystometric capacity; Max CC—maximum cystometric capacity; Comp—the compliance of the bladder wall; EMG 1—electromyography of sphincter at the beginning of the filling phase; EMG 2—electromyography of sphincter at the end of the filling phase.

**Table 3:** Correlations between serum biochemical parameters and RBP4 and RBP4/creatinine ratio among MMC patients. * *p* < 0.05.

	Screat (mg/dL)	SCysC (mg/L)	SUA (mg/dL)	TG (mg/dL)	TChol (mg/dL)	HDL (mg/dL)	LDL (mg/dL)
RBP4 (ng/mL)	−0.17	−0.076	−0.115	−0.315	−0.106	0.308	−0.58 *
RBP4/creat (ng/mg)	−0.253	0.158	−0.126	−0.409	−0.268	0.424	−0.473
Screat (mg/dL)	x	−0.023	0.499 *	0.178	−0.337 *	−0.328	−0.314
SCysC (mg/L)	x	x	0.439 *	0.17	0.085	0.12	−0.012
SUA (mg/dL)	x	x	x	0.742	0.267	−0.699	0.313

Screat—serum creatinine; SCysC—serum cystatin C; SUA—serum uric acid; TG—triglycerides; TChol—total cholesterol; HDL—high-density lipoprotein; LDL—low-density lipoprotein; RBP4—retinol binding protein 4; RBP4/creat—RBP4/creatinine ratio.

**Table 4:** Correlations between urodynamic parameters and renal function parameters and Hoffer’s scale in patients with neurogenic bladder after MMC. * *p* < 0.05.

GFR	PVR	VV	Qavr	Qmax	TV	TQ	Tdel	TQmax	Comp	CC	Pdet CC	HS	
0.130	0.518 *	−0.643 *	−0.100	−0.429	−0.176	−0.141	0.383	0.123	0.022	−0.008	0.193	−0.261	RBP4
0.326 *	0.620 *	−0.392 *	−0.485	−0.442	−0.271	−0.307	0.459 *	0.064	−0.205	−0.073	0.178	−0.612 *	RBP4/creat
−0.013	0.677	−0.521	−0.492	−0.376	−0.376	−0.376	0.900 *	−0.405	−0.219	0.048	x	−0.225	P det CC
−0.298	0.030	0.257	0.142	0.314	−0.371	−0.371	0.601	−0.428	0.771 *	x	0.048	−0.052	CC
−0.373	−0.819 *	0.371	0.085	0.142	−0.085	−0.085	0.204	−0.314	x	0.771	−0.219	0.009	Comp
0.173	0.250	−0.065	0.251	−0.275	0.613 *	0.613 *	0.223	x	−0.314	−0.429	−0.406	0.029	TQ max
−0.229	0.722 *	−0.540	−0.600	−0.114 *	0.234	0.130	x	0.223	0.200	0.600	0.900 *	−0.495 *	T del
−0.154	0.037	0.329	0.028	−0.009	0.965 *	x	0.131	0.618 *	−0.085	−0.371	−0.377	0.079	TQ
−0.255	0.114	0.292	−0.024	−0.079	x	0.964 *	0.234	0.637 *	−0.086	−0.371	−0.376	0.039	TV
0.158	−0.690 *	0.592 *	0.942 *	x	−0.079	−0.009	−0.715 *	−0.275	0.142	0.314	−0.378	0.412	Q max
0.300	−0.394	0.885 *	x	0.942 *	0.028	0.021	−0.680	0.257	0.085	0.143	−0.493	0.507	Q avr
0.048	−0.693 *	x	0.885	0.592 *	0.292	0.329	−0.540 *	−0.065	0.371	0.257	−0.522	0.412	VV
−0.109	x	−0.693 *	−0.394	−0.690 *	0.114	0.037	0.722 *	0.259	−0.819 *	0.039	0.678	−0.588 *	PVR

GFR—glomerular filtration rate; PVR—post-void residual; VV—voided volume; Qavr—average flow rate; Qmax—maximum flow rate; TV—voiding time; TQ—flow time; T del—delay time; TQmax—time to maximum flow rate; Comp—the compliance of the bladder wall; CC—cystometric capacity; P det CC—the detrusor pressure at maximum cystometric capacity; RBP4—retinol binding protein 4; RBP4/creat—RBP4/creatinine ratio.

## Data Availability

The data presented in this study are available on request from the corresponding author. The data are not publicly available for ethical and privacy reasons.
